# Best practice data life cycle approaches for the life sciences

**DOI:** 10.12688/f1000research.12344.2

**Published:** 2018-06-04

**Authors:** Philippa C. Griffin, Jyoti Khadake, Kate S. LeMay, Suzanna E. Lewis, Sandra Orchard, Andrew Pask, Bernard Pope, Ute Roessner, Keith Russell, Torsten Seemann, Andrew Treloar, Sonika Tyagi, Jeffrey H. Christiansen, Saravanan Dayalan, Simon Gladman, Sandra B. Hangartner, Helen L. Hayden, William W.H. Ho, Gabriel Keeble-Gagnère, Pasi K. Korhonen, Peter Neish, Priscilla R. Prestes, Mark F. Richardson, Nathan S. Watson-Haigh, Kelly L. Wyres, Neil D. Young, Maria Victoria Schneider

**Affiliations:** 1 EMBL Australia Bioinformatics Resource, The University of Melbourne, Parkville, VIC, 3010, Australia; 2 Melbourne Bioinformatics, The University of Melbourne, Parkville, VIC, 3010, Australia; 3 NIHR BioResource, University of Cambridge and Cambridge University Hospitals NHS Foundation Trust Hills Road, Cambridge , CB2 0QQ, UK; 4 Australian National Data Service, Monash University, Malvern East , VIC, 3145, Australia; 5 Lawrence Berkeley National Laboratory, Environmental Genomics and Systems Biology Division, Berkeley, CA, 94720, USA; 6 European Bioinformatics Institute (EMBL-EBI), European Molecular Biology Laboratory, Cambridge, CB10 1SD, UK; 7 School of BioSciences, The University of Melbourne, Parkville, VIC, 3010, Australia; 8 Metabolomics Australia, School of BioSciences, The University of Melbourne, Parkville, VIC, 3010, Australia; 9 Australian Genome Research Facility Ltd, Parkville, VIC, 3052, Australia; 10 Monash Bioinformatics Platform, Monash University, Clayton, VIC, 3800, Australia; 11 Queensland Cyber Infrastructure Foundation and the University of Queensland Research Computing Centre, St Lucia, QLD, 4072, Australia; 12 School of Biological Sciences, Monash University, Clayton, VIC, 3800, Australia; 13 Agriculture Victoria, AgriBio, Centre for AgriBioscience, Department of Economic Development, Jobs, Transport and Resources (DEDJTR), Bundoora, VIC, 3083, Australia; 14 Faculty of Veterinary and Agricultural Sciences, The University of Melbourne, Parkville, VIC, 3010, Australia; 15 The University of Melbourne, Parkville, VIC, 3010, Australia; 16 Faculty of Science and Engineering, Federation University Australia, Mt Helen , VIC, 3350, Australia; 17 Bioinformatics Core Research Group & Centre for Integrative Ecology, Deakin University, Geelong, VIC, 3220, Australia; 18 School of Agriculture, Food and Wine, University of Adelaide, Glen Osmond, SA, 5064, Australia; 19 Department of Biochemistry and Molecular Biology, Bio21 Molecular Science and Biotechnology Institute, The University of Melbourne, Parkville, VIC, 3010, Australia

**Keywords:** data sharing, data management, open science, bioinformatics, reproducibility

## Abstract

Throughout history, the life sciences have been revolutionised by technological advances; in our era this is manifested by advances in instrumentation for data generation, and consequently researchers now routinely handle large amounts of heterogeneous data in digital formats. The simultaneous transitions towards biology as a data science and towards a ‘life cycle’ view of research data pose new challenges. Researchers face a bewildering landscape of data management requirements, recommendations and regulations, without necessarily being able to access data management training or possessing a clear understanding of practical approaches that can assist in data management in their particular research domain.

Here we provide an overview of best practice data life cycle approaches for researchers in the life sciences/bioinformatics space with a particular focus on ‘omics’ datasets and computer-based data processing and analysis. We discuss the different stages of the data life cycle and provide practical suggestions for useful tools and resources to improve data management practices.

## Introduction

Technological data production capacity is revolutionising biology ^1^, but is not necessarily correlated with the ability to efficiently analyse and integrate data, or with enabling long-term data sharing and reuse. There are selfish as well as altruistic benefits to making research data reusable ^2^: it allows one to find and reuse one’s own previously-generated data easily; it is associated with higher citation rates ^3, 4^; and it ensures eligibility for funding from and publication in venues that mandate data sharing, an increasingly common requirement (e.g. Final NIH statement on sharing research data, Wellcome Trust policy on data management and sharing, Bill & Melinda Gates Foundation open access policy). Currently we are losing data at a rapid rate, with up to 80% unavailable after 20 years ^5^. This affects reproducibility - assessing the robustness of scientific conclusions by ensuring experiments and findings can be reproduced - which underpins the scientific method. Once access to the underlying data is lost, replicability, reproducibility and extensibility ^6^ are reduced.

**Figure 1.**
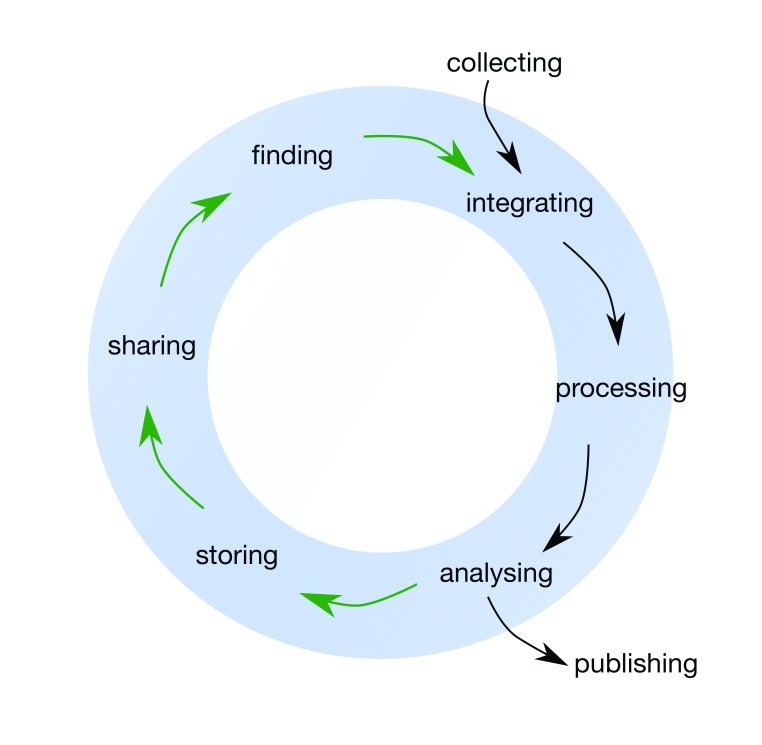
The Data Life Cycle framework for bioscience, biomedical and bioinformatics data that is discussed throughout this article. Black arrows indicate the ‘traditional’, linear view of research data; the green arrows show the steps necessary for data reusability. This framework is likely to be a simplified representation of any given research project, and in practice there would be numerous ‘feedback loops’ and revisiting of previous stages. In addition, the publishing stage can occur at several points in the data life cycle.

At a broader societal level, the full value of research data may go beyond the initial use case in unforeseen ways ^7, 8^, so ensuring data quality and reusability is crucial to realising its potential value ^9– 12^. The recent publication of the FAIR principles ^9, 13^ identifies four key criteria for high-quality research data: the data should be Findable, Accessible, Interoperable and Reusable. Whereas a traditional view of data focuses on collecting, processing, analysing data and publishing results only, a life cycle view reveals the additional importance of finding, storing and sharing data ^11^. Throughout this article, we present a researcher-focused data life cycle framework that has commonalities with other published frameworks [e.g. the DataONE Data Life Cycle, the US geological survey science data lifecycle model and ^11, 14– 15^], but is aimed at life science researchers specifically ( Figure 1).

Learning how to find, store and share research data is not typically an explicit part of undergraduate or postgraduate training in the biological sciences ^16– 18^, though some subdomains (e.g. ecology) have a history of data management advice ^8, 19^. The scope, size and complexity of datasets in many fields has increased dramatically over the last 10–20 years, but the knowledge of how to manage this data is currently limited to specific cohorts of ‘information managers’ (e.g. research data managers, research librarians, database curators and IT professionals with expertise in databases and data schemas ^18^). In response to institutional and funding requirements around data availability, a number of tools and educational programs have been developed to help researchers create Data Management Plans to address elements of the data lifecycle ^20^; however, even when a plan is mandated, there is often a gap between the plan and the actions of the researcher ^10^.

This publication targets life science researchers wanting to improve their data management practice but will also be relevant to life science journals, funders, and research infrastructure bodies. It arose from a 2016 workshop series on the data lifecycle for life science researchers run by EMBL Australia Bioinformatics Resource ^21^, which provided opportunities to (i) map the current approaches to the data life cycle in biology and bioinformatics, and (ii) present and discuss best practice approaches and standards for key international projects with Australian life scientists and bioinformaticians. Throughout the article we highlight some specific data management challenges mentioned by participants. An earlier version of this article can be found on bioRxiv ( https://doi.org/10.1101/167619).

## Finding data

In biology, research data is frequently published as supplementary material to articles, on personal or institutional websites, or in non-discipline-specific repositories like Figshare and Dryad ^22^. In such cases, data may exist behind a paywall, there is no guarantee it will remain extant, and, unless one already knows it exists and its exact location, it may remain undiscovered ^23^. It is only when a dataset is added to a public data repository, along with accompanying standardized descriptive metadata (see ***Collecting data***), that it can be indexed and made publicly available ^24^. Data repositories also provide unique identifiers that increase findability by enabling persistent linking from other locations and permanent association between data and its metadata.

In the field of molecular biology, a number of bioinformatics-relevant organisations host public data repositories. National and international-level organisations of this kind include the European Bioinformatics Institute (EMBL-EBI) ^25^, the National Centre for Biotechnology Information (NCBI) ^26^, the DNA Data Bank of Japan (DDBJ) ^27^, the Swiss Institute of Bioinformatics (SIB) ^28^, and the four data center members of the worldwide Protein Data Bank ^29^, which mirror their shared data with regular, frequent updates. This shared central infrastructure is hugely valuable to research and development. For example, EMBL-EBI resources have been valued at over £270 million per year and contribute to ~£1 billion in research efficiencies; a 20-fold return on investment ^30^.

Numerous repositories are available for biological data (see Table 1 for an overview), though repositories are still lacking for some data types and sub-domains ^31^. Due to privacy regulations, human data is generally not freely available and these repositories typically require access requests on an individual dataset basis ^32, 33^. Tools like the dbGAP browser ^34^ and the Beacon Network ^35^ can assist in identifying relevant limited-access datasets and reduce the burden associated with requesting and downloading data.

Many specialised data repositories exist outside of the shared central infrastructure mentioned, often run voluntarily or with minimal funding. Support for biocuration, hosting and maintenance of these smaller-scale but key resources is a pressing problem ^36– 38^. The quality of the user-submitted data in public repositories ^39, 40^ can mean that public datasets require extra curation before reuse. Unfortunately, due to low uptake of established methods (see the EMBL-EBI and NCBI third-party annotation policies; ^41^) to correct the data ^40^, the results of extra curation may not find their way back into the repositories. Repositories are often not easily searched by generic web search engines ^31^. Registries, which form a secondary layer linking multiple, primary repositories, may offer a more convenient way to search across multiple repositories for data relevant to a researcher’s topics of interest ^42^.

**Table 1.** Overview of some representative databases, registries and other tools to find life science data. A more complete list can be found at FAIRsharing.

Database/ registry	Name	Description	Datatypes	URL
Database	Gene Ontology	Repository of functional roles of gene products, including: proteins, ncRNAs, and complexes.	Functional roles as determined experimentally or through inference. Includes evidence for these roles and links to literature	http://geneontology.org/
Database	Kyoto Encyclopedia of Genes and Genomes (KEGG)	Repository for pathway relationships of molecules, genes and cells, especially molecular networks	Protein, gene, cell, and genome pathway membership data	http://www.genome.jp/kegg/
Database	OrthoDB	Repository for gene ortholog information	Protein sequences and orthologous group annotations for evolutionarily related species groups	http://www.orthodb.org/
Database with analysis layer	eggNOG	Repository for gene ortholog information with functional annotation prediction tool	Protein sequences, orthologous group annotations and phylogenetic trees for evolutionarily related species groups	http://eggnogdb.embl.de/
Database	European Nucleotide Archive (ENA)	Repository for nucleotide sequence information	Raw next-generation sequencing data, genome assembly and annotation data	http://www.ebi.ac.uk/ena
Database	Sequence Read Archive (SRA)	Repository for nucleotide sequence information	Raw high-throughput DNA sequencing and alignment data	https://www.ncbi.nlm.nih.gov/sra/
Database	GenBank	Repository for nucleotide sequence information	Annotated DNA sequences	https://www.ncbi.nlm.nih.gov/genbank/
Database	ArrayExpress	Repository for genomic expression data	RNA-seq, microarray, CHIP-seq, Bisulfite-seq and more (see https://www.ebi.ac.uk/arrayexpress/ help/experiment_types.html for full list)	https://www.ebi.ac.uk/arrayexpress/
Database	Gene Expression Omnibus (GEO)	Repository for genetic/genomic expression data	RNA-seq, microarray, real-time PCR data on gene expression	https://www.ncbi.nlm.nih.gov/geo/
Database	PRIDE	Repository for proteomics data	Protein and peptide identifications, post-translational modifications and supporting spectral evidence	https://www.ebi.ac.uk/pride/archive/
Database	Protein Data Bank (PDB)	Repository for protein structure information	3D structures of proteins, nucleic acids and complexes	https://www.wwpdb.org/
Database	MetaboLights	Repository for metabolomics experiments and derived information	Metabolite structures, reference spectra and biological characteristics; raw and processed metabolite profiles	http://www.ebi.ac.uk/metabolights/
Ontology/ database	ChEBI	Ontology and repository for chemical entities	Small molecule structures and chemical properties	https://www.ebi.ac.uk/chebi/
Database	Taxonomy	Repository of taxonomic classification information	Taxonomic classification and nomenclature data for organisms in public NCBI databases	https://www.ncbi.nlm.nih.gov/taxonomy
Database	BioStudies	Repository for descriptions of biological studies, with links to data in other databases and publications	Study descriptions and supplementary files	https://www.ebi.ac.uk/biostudies/
Database	Biosamples	Repository for information about biological samples, with links to data generated from these samples located in other databases	Sample descriptions	https://www.ebi.ac.uk/biosamples/
Database with analysis layer	IntAct	Repository for molecular interaction information	Molecular interactions and evidence type	http://www.ebi.ac.uk/intact/
Database	UniProtKB (SwissProt and TrEMBL)	Repository for protein sequence and function data. Combines curated (UniProtKB/SwissProt) and automatically annotated, uncurated (UniProtKB/TrEMBL) databases	Protein sequences, protein function and evidence type	http://www.uniprot.org/
Database	European Genome- Phenome Archive	Controlled-access repository for sequence and genotype experiments from human participants whose consent agreements authorise data release for specific research use	Raw, processed and/or analysed sequence and genotype data along with phenotype information	https://www.ebi.ac.uk/ega/
Database with analysis layer	EBI Metagenomics	Repository and analysis service for metagenomics and metatranscriptomics data. Data is archived in ENA	Next-generation sequencing metagenomic and metatranscriptomic data; metabarcoding (amplicon-based) data	https://www.ebi.ac.uk/metagenomics/
Database with analysis layer	MG-RAST	Repository and analysis service for metagenomics data.	Next-generation sequencing metagenomic and metabarcoding (amplicon-based) data	http://metagenomics.anl.gov/
Registry	Omics DI	Registry for dataset discovery that currently spans 11 data repositories: PRIDE, PeptideAtlas, MassIVE, GPMDB, EGA, Metabolights, Metabolomics Workbench, MetabolomeExpress, GNPS, ArrayExpress, ExpressionAtlas	Genomic, transcriptomic, proteomic and metabolomic data	http://www.omicsdi.org
Registry	DataMed	Registry for biomedical dataset discovery that currently spans 66 data repositories	Genomic, transcriptomic, proteomic, metabolomic, morphology, cell signalling, imaging and other data	https://datamed.org
Registry	Biosharing	Curated registry for biological databases, data standards, and policies	Information on databases, standards and policies including fields of research and usage recommendations by key organisations	https://biosharing.org/
Registry	re3data	Registry for research data repositories across multiple research disciplines	Information on research data repositories, terms of use, research fields	http://www.re3data.org

## Collecting data

The most useful data has associated information about its creation, its content and its context - called metadata. If metadata is well structured, uses consistent element names and contains element values with specific descriptions from agreed-upon vocabularies, it enables machine readability, aggregation, integration and tracking across datasets: allowing for Findability, Interoperability and Reusability ^9, 31^. One key approach in best-practice metadata collection is to use controlled vocabularies built from ontology terms. Biological ontologies are tools that provide machine-interpretable representations of some aspect of biological reality ^31, 43^. They are a way of organising and defining objects (i.e. physical entities or processes), and the relationships between them. Sourcing metadata element values from ontologies ensures that the terms used in metadata are consistent and clearly defined. There are several user-friendly tools available to assist researchers in accessing, using and contributing to ontologies ( Table 2).

**Table 2.** Useful ontology tools to assist in metadata collection.

Tool	Task	URL
Ontology Lookup Service	Discover different ontologies and their contents	http://www.ebi.ac.uk/ols/
OBO Foundry	Table of open biomedical ontologies with information on development status, license and content	http://obofoundry.org/
Zooma	Assign ontology terms using curated mapping	http://www.ebi.ac.uk/spot/zooma/
Webulous	Create new ontology terms easily	https://www.ebi.ac.uk/efo/webulous/
Ontobee	A linked data server that facilitates ontology data sharing, visualization, and use.	http://www.ontobee.org

Adopting standard data and metadata formats and syntax is critical for compliance with FAIR principles ^9, 24, 31, 42, 44^. Biological and biomedical research has been considered an especially challenging research field in this regard, as datatypes are extremely heterogeneous and not all have defined data standards ^44, 45^; many existing data standards are complex and therefore difficult to use ^45^, or only informally defined, and therefore subject to variation, misrepresentation, and divergence over time ^44^. Nevertheless, well-established standards exist for a variety of biological data types ( Table 3). FAIRsharing is a useful registry of data standards and policies that also indicates the current status of standards for different data types and those recommended by databases and research organisations ^42^.

**Table 3.** Overview of common standard data formats for ‘omics data. A more complete list can be found at FAIRsharing.

Data type	Format name	Description	Reference or URL for format specification	URLs for repositories accepting data in this format
Raw DNA/RNA sequence	FASTA FASTQ HDF5 SAM/BAM/ CRAM	FASTA is a common text format to store DNA/RNA/Protein sequence and FASTQ combines base quality information with the nucleotide sequence. HDF5 is a newer sequence read formats used by long read sequencers e.g. PacBio and Oxford Nanopore. Raw sequence can also be stored in unaligned SAM/BAM/ CRAM format	74 75 https://support.hdfgroup.org/HDF5/ https://samtools.github.io/hts-specs/ https://www.ncbi.nlm.nih.gov/ sra/docs/submitformats/ http://www.ebi.ac.uk/ena/ submit/data-formats
Assembled DNA sequence	FASTA Flat file AGP	Assemblies without annotation are generally stored in FASTA format. Annotation can be integrated with assemblies in contig, scaffold or chromosome flat file format. AGP files are used to describe how smaller fragments are placed in an assembly but do not contain the sequence information themselves	41 http://www.ebi.ac.uk/ena/submit/contig-flat-file http://www.ebi.ac.uk/ena/submit/scaffold-flat-file https://www.ncbi.nlm.nih.gov/assembly/agp/AGP_ Specification/	http://www.ebi.ac.uk/ena /submit/genomes-sequence- submission
Aligned DNA sequence	SAM/BAM CRAM	Sequences aligned to a reference are represented in sequence alignment and mapping format (SAM). Its binary version is called BAM and further compression can be done using the CRAM format	https://samtools.github.io/hts-specs/	https://www.ncbi.nlm.nih.gov/ sra/docs/submitformats/#bam
Gene model or genomic feature annotation	GTF/GFF/ GFF3 BED GB/GBK	General feature format or general transfer format are commonly used to store genomic features in tab-delimited flat text format. GFF3 is a more advanced version of the basic GFF that allows description of more complex features. BED format is a tab-delimited text format that also allows definition of how a feature should be displayed (e.g. on a genome browser). GenBank flat file Format (GB/GBK) is also commonly used but not well standardised	https://github.com/The-Sequence-Ontology/ Specifications/blob/master/gff3.md https://genome.ucsc.edu/FAQ/FAQformat.html https://genome.ucsc.edu/FAQ/FAQformat.html https://www.ncbi.nlm.nih.gov/Sitemap/ samplerecord.html	http://www.ensembl.org/info/ website/upload/gff.html http://www.ensembl.org/info/ website/upload/gff3.html
Gene functional annotation	GAF (GPAD and RDF will also be available in 2018)	A GAF file is a GO Annotation File containing annotations made to the GO by a contributing resource such as FlyBase or Pombase. However, the GAF standard is applicable outside of GO, e.g. using other ontologies such as PO. GAF (v2) is a simple tab-delimited file format with 17 columns to describe an entity (e.g. a protein), its annotation and some annotation metadata	http://geneontology.org/page/go-annotation-file- format-20	http://geneontology.org/page/ submitting-go-annotations
Genetic/genomic variants	VCF	A tab-delimited text format to store meta-information as header lines followed by information about variants position in the genome. The current version is VCF4.2	https://samtools.github.io/hts-specs/VCFv4.2.pdf	http://www.ensembl.org/info/ website/upload/var.html
Interaction data	PSI-MI XML MITAB	Data formats developed to exchange molecular interaction data, related metadata and fully describe molecule constructs	http://psidev.info/groups/molecular-interactions	http://www.ebi.ac.uk/intact
Raw metabolite profile	mzML nmrML	XML based data formats that define mass spectrometry and nuclear magnetic resonance raw data in Metabolomics	http://www.psidev.info/mzml http://nmrml.org/	
Protein sequence	FASTA	A text-based format for representing nucleotide sequences or protein sequences, in which nucleotides or amino acids are represented using single-letter codes	74	www.uniprot.org
Raw proteome profile	mzML	A formally defined XML format for representing mass spectrometry data. Files typically contain sequences of mass spectra, plus metadata about the experiment	http://www.psidev.info/mzml	www.ebi.ac.uk/pride
Organisms and specimens	Darwin Core	The Darwin Core (DwC) standard facilitates the exchange of information about the geographic location of organisms and associated collection specimens	http://rs.tdwg.org/dwc/	

Most public repositories for biological data (see Table 1 and ***Storing data*** section) require that minimum metadata be submitted accompanying each dataset ( Table 4). This minimum metadata specification typically has broad community input ^46^. Minimum metadata standards may not include the crucial metadata fields that give the full context of the particular research project ^46^, so it is important to gather metadata early, understand how to extend a minimum metadata template to include additional fields in a structured way, and think carefully about all the relevant pieces of metadata information that might be required for reuse.

**Table 4.** Some community-designed minimum information criteria for metadata specifications in life sciences. A more complete list can be found at FAIRsharing.

Name	Description	Examples of projects/databases that use this specification	URL
MINSEQE	Minimum Information about a high- throughput SEQuencing Experiment	Developed by the Functional Genomics Data Society. Used in the NCBI Sequence Read Archive, ArrayExpress	http://fged.org/site_media/pdf/MINSEQE_1.0.pdf
MIxS - MIGS/MIMS	Minimum Information about a (Meta)Genome Sequence. The MIMS extension includes key environmental metadata	Developed by the Genomic Standards Consortium. Numerous adopters including NCBI/EBI/DDBJ databases	http://wiki.gensc.org/index.php?title=MIGS/MIMS
MIMARKS	Minimum Information about a MARKer gene Sequence. This is an extension of MIGS/MIMS for environmental sequences	Developed by the Genomic Standards Consortium. Numerous adopters including NCBI/EBI/DDBJ databases	http://wiki.gensc.org/index.php?title=MIMARKS
MIMIx	Minimum Information about a Molecular Interaction eXperiment	Developed by the Proteomics Standards Initiative. Adopted by the IMEx Consortium databases	http://www.psidev.info/mimix
MIAPE	Minimum Information About a Proteomics Experiment	Developed by the Proteomics Standards Initiative. Adopted by PRIDE, World- 2DPAGE and ProteomeXchange databases	http://www.psidev.info/miape
Metabolomics Standards Initiative (MSI) standards	Minimal reporting structures that represent different parts of the metabolomics workflow	Developed by the Metabolomics Standards Initiative (MSI) and the Coordination of Standards in Metabolomics (COSMOS) consortium	http://www.metabolomics-msi.org/
MIRIAM	Minimal Information Required In the Annotation of Models. For annotation and curation of computational models in biology	Initiated by the BioModels.net effort. Adopted by the EBI BioModels database and others	http://co.mbine.org/standards/miriam
MIAPPE	Minimum Information About a Plant Phenotyping Experiment. Covers study, environment, experimental design, sample management, biosource, treatment and phenotype	Adopted by the Plant Phenomics and Genomics Research Data Repository and the Genetic and Genomic Information System (GnpIS)	http://cropnet.pl/phenotypes/wp-content/uploads/2016/04/MIAPPE.pdf
MDM	Minimal Data for Mapping for sample and experimental metadata for pathogen genome-scale sequence data	Developed by the Global Microbial Identifier Initiative and EBI. Complies with EBI ENA database submission requirements	http://www.ebi.ac.uk/ena/submit/pathogen-data
FAANG sample metadata specification	Metadata specification for biological samples derived from animals (animals, tissue samples, cells or other biological materials). Complies with EBI database requirements and BioSamples database formats	Developed and used by the Functional Annotation of Animal Genomes Consortium	https://github.com/FAANG/faang-metadata/blob/master/docs/faang_ sample_metadata.md
FAANG experimental metadata specification	Metadata specification for sequencing and array experiments on animal samples	Developed and used by the Functional Annotation of Animal Genomes Consortium	https://github.com/FAANG/faang-metadata/blob/master/docs/faang_ experiment_metadata.md
FAANG analysis metadata specification	Metadata specification for analysis results	Developed and used by the Functional Annotation of Animal Genomes Consortium. NB no public repository exists for this specific datatype	https://github.com/FAANG/faang-metadata/blob/master/docs/faang_ analysis_metadata.md
SNOMED-CT	Medical terminology and pharmaceutical product standard	Commercial but collaboratively-designed product	http://www.snomed.org/snomed-ct

## Integrating, processing and analysing data

**Figure 2.**
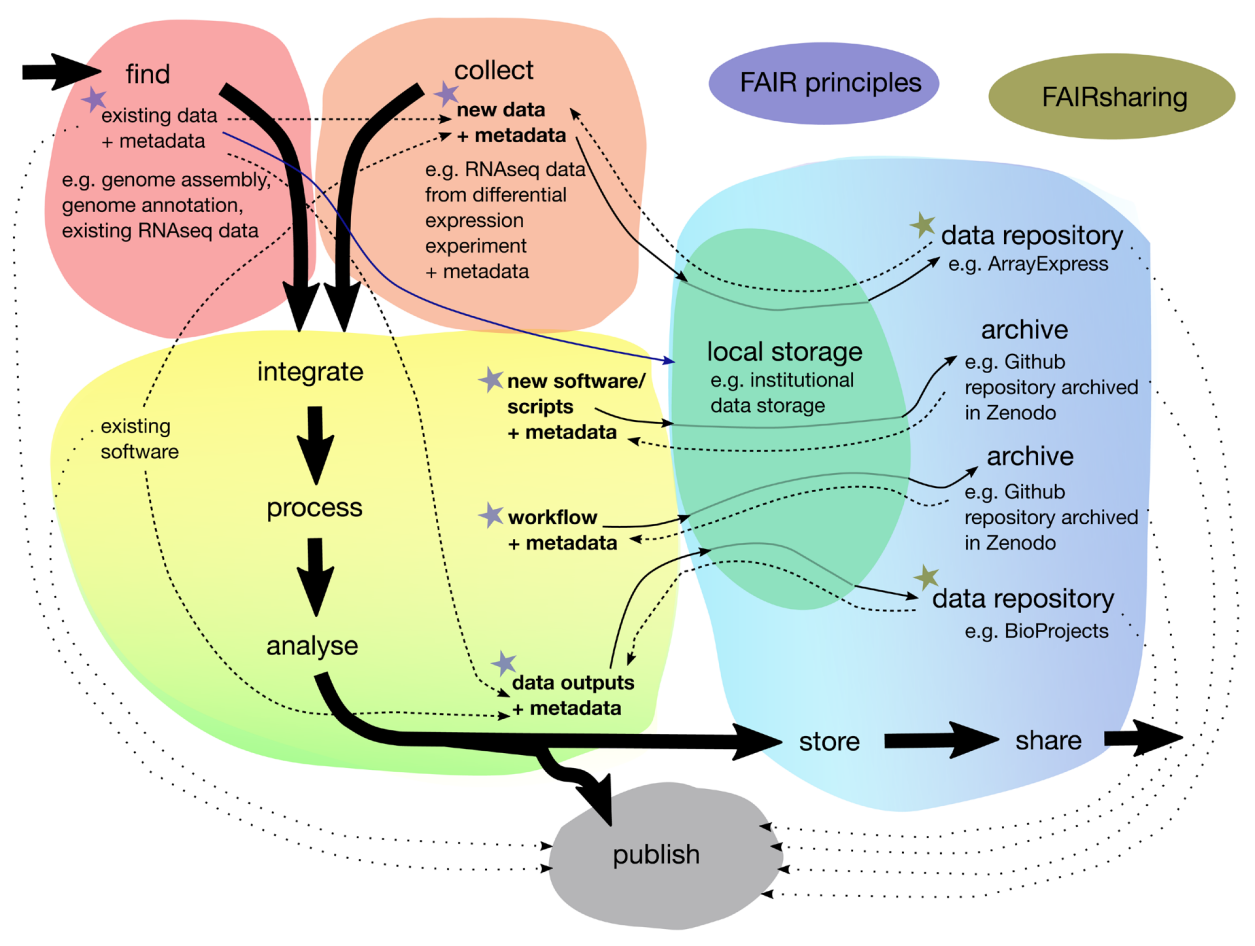
Flowchart of the data life cycle stages applied to an example research project. Bold text indicates new data, software or workflow objects created during the project. Solid thin arrows indicate movement of objects from creation to storage and sharing. Dashed thin arrows indicate where downstream entities should influence decisions made at a given step. (For example, the choice of format, granularity, metadata content and structure of new data collected may be influenced by existing software requirements, existing data characteristics and requirements of the archive where the data will be deposited). Purple stars indicate objects for which the FAIR principles ^9^ can provide further guidance. Dotted thin arrows indicate citation of an object using its unique persistent identifier. Brown stars indicate where FAIRsharing can help identify appropriate archives for storing and sharing.

Where existing and/or newly-collected datasets are to be used in the same experiment, they must first be integrated. This may involve initial processing of one or more datasets so that they share format and granularity, or so that relevant fields map correctly. The researcher also needs to ensure integration at ‘dependency’ level: for example, controlled vocabularies or genome assemblies used in data generation/processing must match or be easily converted. The plethora of autonomous data repositories has created problems with mapping data and annotations among repositories ^47, 48^. Current large-scale efforts aim to improve interoperability using Linked Data and other Semantic Web tools ^48^ as well as extensive ontology development (see ***Collecting data*** section). The Monarch Initiative is an example of a project that achieves new insights by integrating existing data from multiple sources: in this case, data from animal and human genetic, phenotypic and other repositories is brought together via a custom data flow to help identify unrecognised animal models for human disease ^49^. In smaller projects, the need for individual researchers to integrate data will often inform the way new data is collected, to ensure it matches existing datasets, creating a feedback loop in the data lifecycle that highlights the need for prior planning ( Figure 2). Seamless solutions are still some way off ^50^ for all but a handful of applications.

Recording and reporting how research data is processed and analysed computationally is crucial for reproducibility and assessment of research quality ^1, 51^. This can be aided by scientific workflow approaches that facilitate both recording and reproducing processing and analysis steps ^1^, though many experiments will require ‘one-off’ workflows that may not function with existing workflow management systems. Full reproducibility requires access to the software, software versions, workflow, dependencies and operating system used as well as the data and software code itself ^1, 52^. Therefore, although computational work is often seen as enabling reproducibility in the short term, in the long term it is fragile and reproducibility is limited (e.g. discussion by D. Katz, K. Hinsen and C.T. Brown). Best-practice approaches for preserving data processing and analysis code involve hosting source code in a repository where it receives a unique identifier and is under version control; where it is open, accessible, interoperable and reusable - broadly mapping to the FAIR principles for data. Github and Bitbucket, for example, fulfil these criteria, and Zenodo additionally generates Digital Object Identifiers (DOIs) for submissions and guarantees long-term archiving. Workflows can also be preserved in repositories along with relevant annotations (reviewed in 1). A complementary approach is containerised computing (e.g. Docker) which bundles operating system, software, code and potentially workflows and data together. Several recent publications have suggested ways to improve current practice in research software development to aid in reproducibility ^15, 53– 55^.

The same points hold for wet-lab data production: for full reproducibility within and outside the lab, it is important to capture and enable access to specimen cell lines, tissue samples and/or DNA as well as reagents ^56^. Wet-lab methods can be captured in electronic laboratory notebooks and reported in the Biosamples database ^57^, protocols.io or OpenWetWare; specimens can be lodged in biobanks, culture or museum collections ^58– 62^; but the effort involved in enabling full reproducibility remains extensive. Electronic laboratory notebooks are frequently suggested as a sensible way to make this information openly available and archived ^63^. Some partial solutions exist (e.g. LabTrove, BlogMyData, Benchling and others ^64^), including tools for specific domains such as the Scratchpad Virtual Research Environment for natural history research ^65^. Other tools can act as or be combined to produce notebooks for small standalone code-based projects (see 66 and update), including Jupyter Notebook, Rmarkdown, and Docker. However, it remains a challenge to implement online laboratory notebooks to cover both field/lab work and computer-based work, especially when computer work is extensive, involved and non-modular ^51^. Currently, no best-practice guidelines or minimum information standards exist for use of electronic laboratory notebooks ^6^. We suggest that appropriate minimum information to be recorded for most computer-based tasks should include date, task name and brief description, aim, actual command(s) used, software names and versions used, input/output file names and locations, script names and locations, all in a simple text format.

In the authors’ experience, the data processing and analysis stage is one of the most challenging for openness. As reported elsewhere ^16– 18^, we have observed a gap between modern biological research as a field of data science, and biology as it is still mostly taught in undergraduate courses, with little or no focus on computational analysis, or project or data management. This gap has left researchers lacking key knowledge and skills required to implement best practices in dealing with the life cycle of their data.

## Publishing data

Traditionally, scientific publications included raw research data, but in recent times datasets have grown beyond the scope of practical inclusion in a manuscript ^11, 51^. Selected data outputs are often included without sharing or publishing the underlying raw data ^14^. Journals increasingly recommend or require deposition of raw data in a public repository [e.g. 67], although exceptions have been made for publications containing commercially-relevant data ^68^. The current data-sharing mandate is somewhat field-dependent ^5, 69^ and also varies within fields ^70^. For example, in the field of bioinformatics, the UPSIDE principle ^71^ is referred to by some journals (e.g. Bioinformatics), while others have journal- or publisher-specific policies (e.g. BMC Bioinformatics).

The vast majority of scientific journals require inclusion of processing and analysis methods in ‘sufficient detail for reproduction’ (e.g. Public Library of Science submission and data availability guidelines; International Committee of Medical Journal Editors manuscript preparation guidelines; Science instructions for authors; Elsevier Cell Press STAR Methods; and ^72^), though journal requirements are diverse and complex ^73^, and the level of detail authors provide can vary greatly in practice ^76, 77^. More recently, many authors have highlighted that full reproducibility requires sharing data and resources at all stages of the scientific process, from raw data (including biological samples) to full methods and analysis workflows ^1, 6, 61, 77^. However, this remains a challenge ^78, 79^, as discussed in the ***Processing and analysing data*** section. To our knowledge, strategies for enabling computational reproducibility are currently not mandated by any scientific journal.

A recent development in the field of scientific publishing is the establishment of ‘data journals’: scientific journals that publish papers describing datasets. This gives authors a vehicle to accrue citations (still a dominant metric of academic impact) for data production alone, which can often be labour-intensive and expensive yet is typically not well recognised under the traditional publishing model. Examples of this article type include the Data Descriptor in Scientific Data and the Data Note in GigaScience, which do not include detailed new analysis but rather focus on describing and enabling reuse of datasets.

The movement towards sharing research publications themselves (‘Open Access Publishing’) has been discussed extensively elsewhere [e.g. 23, 80, 81]. Publications have associated metadata (creator, date, title etc.; see Dublin Core Metadata Initiative metadata terms) and unique identifiers (PubMed ID for biomedical and some life science journals, DOIs for the vast majority of journals; see Table 5). The ORCID system enables researchers to claim their own unique identifier, which can be linked to their publications. The use of unique identifiers within publications referring to repository records (e.g. genes, proteins, chemical entities) is not generally mandated by journals, although it would ensure a common vocabulary is used and so make scientific results more interoperable and reusable ^82^. Some efforts are underway to make this easier for researchers: for example, Genetics and other Genetics Society of America journals assist authors in linking gene names to model organism database entries.

**Table 5.** Identifiers throughout the data life cycle.

Name	Relevant stage of data life cycle	Description	URL
Digital Object Identifier (DOI)	Publishing, Sharing, Finding	A unique identifier for a digital (or physical or abstract) object	https://www.doi.org/
Open Researcher and Contributor ID (ORCID)	Publishing	An identifier for a specific researcher that persists across publications and other research outputs	https://orcid.org/
Repository accession number	Finding, Processing/ Analyzing, Publishing, Sharing, Storing	A unique identifier for a record within a repository. Format will be repository-specific. Examples include NIH UIDs (unique identifiers) and accession numbers; ENA accession numbers; PDB IDs	For example, https://support.ncbi.nlm.nih.gov/link/portal/28045/28049/ Article/499/ http://www.ebi.ac.uk/ena/submit/accession-number-formats
Pubmed ID (PMID)	Publishing	An example of a repository-specific unique identifier: PubMed IDs are used for research publications indexed in the PubMed database	https://www.ncbi.nlm.nih.gov/pubmed/
International Standard Serial Number (ISSN)	Publishing	A unique identifier for a journal, magazine or periodical	http://www.issn.org/
International Standard Book Number (ISBN)	Publishing	A unique identifier for a book, specific to the title, edition and format	https://www.isbn-international.org

## Storing data

While primary data archives are the best location for raw data and some downstream data outputs ( Table 1), researchers also need local data storage solutions during the processing and analysis stages. Data storage requirements vary among research domains, with major challenges often evident for groups working on taxa with large genomes (e.g. crop plants), which require large storage resources, or on human data, where privacy regulations may require local data storage, access controls (e.g. the GA4GH Security Technology Infrastructure document) and conversion to non-identifiable data if data is to be shared (see ***Sharing data*** section). For data where privacy is a concern, one approach is separating the data storage from the analysis location and limiting the analysis outputs to ‘nondisclosive’ results ^83^. An example is DataShield ^83^, which is mostly used for public health rather than ‘omics’ data. Subdomain-specific practice should be considered when choosing appropriate formats and linking metadata, as outlined in 84. In addition, long-term preservation of research data should consider threats such as storage failure, mistaken erasure, bit rot, outdated media, outdated formats, loss of context and organisational failure ^85^.

## Sharing data

The best-practice approach to sharing biological data is to deposit it (with associated metadata) in a primary archive suitable for that datatype ^8^ that complies with FAIR principles. As highlighted in the ***Storing data*** section, these archives assure both data storage and public sharing as their core mission, making them the most reliable location for long-term data storage. Alternative data sharing venues (e.g. FigShare, Dryad) do not require or implement specific metadata or data standards. This means that while these venues have a low barrier to entry for submitters, the data is not FAIR unless submitters have independently decided to comply with more stringent criteria. If available, an institutional repository may be a good option if there is no suitable archive for that datatype.

Data with privacy concerns (for example, containing human-derived, commercially-important or sensitive environmental information) can require extensive planning and compliance with a range of institutional and regulatory requirements as well as relevant laws ^86^ (for the Australian context, see the Australian National Data Service Publishing and Sharing Sensitive Data Guide, the National Health and Medical Research Council statement on ethical conduct in human research, and the Australian National Medical Research Storage Facility discussion paper on legal, best practice and security frameworks). In particular, it is often necessary for users of the data to be correctly identified, and to subsequently be authenticated via a mechanism such as OpenID, eduGAIN, or (in the Australian context), AAF, which places the onus on ensuring users are correctly identified with institutions that issue their credentials. Knowing who the users are can be used to restrict access, require compliance with the conditions under which the data is provided, and track user activity as an audit trail. The Data Access Compliance Office of the International Cancer Genome Consortium is an example of how to manage requests for access to controlled data. Large-scale collaborations such as the Global Alliance for Genomics and Health (GA4GH) are leading the way in approaches to sharing sensitive data across institutions and jurisdictions ( 87; also see the GA4GH Privacy and Security Policy). Importantly, plans for data sharing should be made at the start of a research project and reviewed during the project, to ensure ethical approval is in place and that the resources and metadata needed for effective sharing are available at earlier stages of the data life cycle ^3^.

In our experience, the majority of life science researchers are familiar with at least some public primary data repositories, and many have submitted data to them previously. A common complaint is around usability of current data submission tools and a lack of transparency around metadata requirements and the rationale for them. Some researchers raise specific issues about the potential limitations of public data repositories where their data departs from the assumptions of the repository (e.g. unusual gene models supported by experimental evidence can be rejected by the automated NCBI curation system). In such cases, researchers can provide feedback to the repositories to deal with such situations, but may not be aware of this - it could be made clearer on the repository websites. Again, this points in part to existing limitations in the undergraduate and postgraduate training received by researchers, where the concepts presented in this article are presented as afterthoughts, if at all. On the repository side, while there is a lot of useful information and training material available to guide researchers through the submission process (e.g. the EMBL-EBI Train Online webinars and online training modules), it is not always linked clearly from the database portals or submission pages themselves. Similarly, while there are specifications and standards available for many kinds of metadata [ Table 4; also see FAIRsharing], many do not have example templates available, which would assist researchers in implementing the standards in practice.

## What can the research community do to encourage best-practice?

We believe that the biological/biomedical community and individual researchers have a responsibility to the public to help advance knowledge by making research data FAIR for reuse ^9^, especially if the data were generated using public funding. There are several steps that can assist in this mission:

1. **Researchers reusing any data should openly acknowledge this fact and fully cite the dataset, using unique identifiers** ^8, 10, 31^.
2. **Researchers should endeavour to improve their own data management practices in line with best practice in their subdomain** – even incremental improvement is better than none!
3. **Researchers should provide feedback** to their institution, data repositories and bodies responsible for community resources (data standards, controlled vocabularies etc.) **where they identify roadblocks** to good data management.
4. **Senior scientists should lead by example** and ensure all the data generated by their laboratories is well-managed, fully annotated with the appropriate metadata and made publicly available in an appropriate repository.
5. **The importance of data management and benefits of data reuse should be taught** at the undergraduate and postgraduate levels ^18^. Computational biology and bioinformatics courses in particular should include material about data repositories, data and metadata standards, data discovery and access strategies. Material should be domain-specific enough for students to attain learning outcomes directly relevant to their research field.
6. Funding bodies are already taking a lead role in this area by requiring the incorporation of a data management plan into grant applications. A next step would be for a **formal check, at the end of the grant period, that this plan has been adhered to and data is available in an appropriate format for reuse** ^10^.
7. **Funding bodies and research institutions should judge quality dataset generation as a valued metric when evaluating grant or promotion applications.**
8. **Similarly, leadership and participation in community efforts in data and metadata standards, and open software and workflow development should be recognised as academic outputs.**
9. **Data repositories should ensure that the data deposition and third-party annotation processes are as FAIR and painless as possible** to the naive researcher, without the need for extensive bioinformatics support ^40^.
10. **Journals should require editors and reviewers to check manuscripts to ensure that all data, including research software code and samples where appropriate, have been made publicly available in an appropriate repository**, and that methods have been described in enough detail to allow re-use and meaningful reanalysis ^8^.

## Conclusions

While the concept of a life cycle for research data is appealing from an Open Science perspective, challenges remain for life science researchers to put this into practice. Among attendees of the workshop series that gave rise to this publication, we noted limited awareness among attendees of the resources available to researchers that assist in finding, collecting, processing, analysis, publishing, storing and sharing FAIR data. We believe this article provides a useful overview of the relevant concepts and an introduction to key organisations, resources and guidelines to help researchers improve their data management practices.

Furthermore, we note that data management in the era of biology as a data science is a complex and evolving topic and both best practices and challenges are highly domain-specific, even within the life sciences. This factor may not always be appreciated at the organisational level, but has major practical implications for the quality and interoperability of shared life science data. Finally, domain-specific education and training in data management would be of great value to the life science research workforce, and we note an existing gap at the undergraduate, postgraduate and short course level in this area.
