# Supplementary material for: Best practice data life cycle approaches for the life sciences
Source: F1000Res. 2018 Jun 4;6:1618. Originally published 2017 Aug 31. [Version 2] doi: 10.12688/f1000research.12344.2 (PMC6069748; doi:10.12688/f1000research.12344.2)
Supplement: Peer review history

# Peer review: Referee response for version 2

**DOI:** 10.5256/f1000research.16485.r34683

**Published:** 2018-07-30

**Authors:** Sven Nahnsen

**Affiliations:** 1 Quantitative Biology Center (QBiC), University of Tübingen, Tübingen, Germany

The authors have sufficiently addressed the issues that were raised. I am happy to recommend the article by Griffin *et al.* for indexing.

I have read this submission. I believe that I have an appropriate level of expertise to confirm that it is of an acceptable scientific standard.

# Peer review: Referee response for version 2

**DOI:** 10.5256/f1000research.16485.r34682

**Published:** 2018-06-19

**Authors:** Johannes Starlinger

**Affiliations:** 1 Department of Anesthesiology and Operative Intensive Care Medicine, Charité – Universitätsmedizin Berlin, Berlin, Germany; 2 Department of Computer Science, Humboldt-Universität zu Berlin, Berlin, Germany

The authors have more than adequately addressed all issues raised in the review, both in their answers to the reviewer's comments and in the amendments of the paper. Thank you very much for this very instructive introductory read for data management for life sciences researchers!

I have read this submission. I believe that I have an appropriate level of expertise to confirm that it is of an acceptable scientific standard.

# Peer review: Referee response for version 1

**DOI:** 10.5256/f1000research.13366.r27113

**Published:** 2017-12-15

**Authors:** Sven Nahnsen

**Affiliations:** 1 Quantitative Biology Center (QBiC), University of Tübingen, Tübingen, Germany

The article "Best practice data life cycle approaches for the life sciences", submitted by Griffin *et al.* reports opinions on how to best manage the growing complexity of scientific data in the life sciences.

 The article touches on an extremely important topic that is currently very purely covered in the literature. In fact, data-driven approaches in the biosciences will strongly rely on professional concepts of data management. In brief, I recommend the indexing of the article, as we urgently need stronger awareness of this topic, upon the implementation of some (probably rather minor) changes to the article. The article nicely illustrates the needs in data life cycle management and also suggests best concepts to be followed by researchers. The main content of the article has been compiled based on a workshop that was attended by the authors. At some statements the article reads like the minutes of this meeting; I suggest editing the corresponding paragraphs to avoid the impression of reading meeting minutes.

 I suggest the following issues to be fixed before indexing:

- Figure 1: This illustration is very important and can be used by many readers. I suggest to use figures wherever possible to replace the words such as “finding”, “integrating”, …
- The reference to Figure 1 in the second paragraph states that it illustrates a specific aim to the life sciences. I don’t see which of these points should be specific to the life science, but would rather argue that these principles are rather generic and provides a cycle for business intelligence processes in general. It might also be a good location to reference the DAMA (Data management association internation, dama.org) and specifically to the DAMA Body of Knowledge, which is one of the few references for data management and also data life cycle considerations. Further needed references should hint to the Global Alliance for Genomics and Health (ga4gh.org).
- Page 13: The paragraph on data sharing missing some discussion on authentication issues. I would like see some introduction and discussion to the OpenID concept. Especially for medical data there need to be appropriate mechanisms to trace users, concepts for data privacy and so on. As a best practice use case for these topics, the mechanism from ICGC could be introduced.
- The following paragraph states: “A few workshop participants…”. Rephrase, no meeting minutes..
- I would have loved to see more use cases/examples for the individual best practices. E.g. for the data sharing the ICGC efforts could be described more thoroughly.
- The article would benefit for 2-3 additional figures. I guess it could be a nice figure to illustrate the concept of controlled vocabularies and/or ontologies. While this seems to be trivial for bioinformaticians/computer scientists, it is not that obvious what it means to non-computer scientists; inspiration for figures can also be obtained by the data sharing mechanisms for the Global alliance for Genomics and Health

Minor things:

- The forth paragraph in the introduction starts with “During the week of 24-28 October 2016…”. I suggest either avoiding that paragraph or formulating it differently. The reader should not be reading the meeting minutes.

I have read this submission. I believe that I have an appropriate level of expertise to confirm that it is of an acceptable scientific standard, however I have significant reservations, as outlined above.

# Peer review: Referee response for version 1

**DOI:** 10.5256/f1000research.13366.r27111

**Published:** 2017-11-21

**Authors:** Johannes Starlinger

**Affiliations:** 1 Department of Anesthesiology and Operative Intensive Care Medicine, Charité – Universitätsmedizin Berlin, Berlin, Germany; 2 Department of Computer Science, Humboldt-Universität zu Berlin, Berlin, Germany

The article gives a brief overview of the data life cycle in the life sciences and offers an entry point for accessing relevant information about current approaches to increasing compliance with the FAIR data sharing principles at each step of this life cycle. It expressly targets "life science researchers wanting to improve their data management practice" and is labeled as an *Opinion *article. 

 The article is well written and comfortable to read, and the concise presentation follows a clear structure. While to me as a biomedical data researcher, who may not strictly belong to the target audience, the article provided only little additional insight, I can well see how - as an entry point - the article provides valuable information to its target audience.

 That said, I believe the article needs clarification and some extension in a few places: 

- The list of authors is quite extensive. Please clarify the roles of the authors in conception/conduction/preparation of the manuscript.
- How exactly does the proposed data life cycle differ from related (cited) suggestions, and why? How is it 'aimed at life science researchers specifically'? (Introduction)
- The tabular overviews of existing resources are a nice asset but they are, of course, not exhaustive. Please clarify how the selections of databases/registries, tools, ontologies etc were made for inclusion in the article - and possibly state where to find more complete lists of resources for the life sciences.
- The *integrating *step of the life cycle has no description in the article - even though this is a very intricate step that often has great influence when *collecting* data (e.g., the choice of ontologies to use for describing collected data and metadata will often depend on the ontologies used in re-used (found) data), and, even more, is at the core of making datasets interoperable, i.e., making them integratable with newly collected data.
- In the *processing* step, you make no mention of Scientific Workflows as a means of integrating, processing, and analyzing data. Your first reference (currently cited in a rather different context) would provide a very good hook for this thriving topic that is all about sharing, reproducibility, and reusability of data processing and analysis methods. On the same lines, containerized computing (e.g., Docker) is only very briefly metioned. Even more than with data, using technologies such as these is crucial for ensuring reproducibility over longer periods of time (when software versions of dependencies have changed, web-services have become unavailable, and so forth).
- The section "What can the research community do to encourage best practice?" gives a rather remote, high level view that addresses several different institutional entities - except for the individual researcher within the target audience who actually has to follow the discussed best practices to enable the data life cycle.

Additionally, here are some suggestions for increasing the usefulness and potential impact of the article within the current target audience, and possibly beyond:

- Important interdependecies between the different steps of the life cycle could be mentioned. For instance, the choice of which ontologies to use for metadata and data in the *collection *step will necessarily be influenced by a) the ontologies used in the data found in public repositiories and reused in the current experiment, b) the ontologies mandated by the repositories the data product is to be published in, and c) the ontologies required and used by the (third party, reused) software applied in the processing of the data. These interdependencies often not only put a limit to the choices available regarding the ontologies to be used but also raise a barrier when conversion and mapping between different ontologies is necessary between steps in the life cycle.
- The topic of data privacy is only very briefly touched but fundamental when it comes to sharing and publishing data. It may be out of scope of this article, but a slightly more thorough discussion of the issue would to its importance more justice, I feel.
- An additional figure that maps the best practices enumerated in the text to the rather coarse life cycle shown in Figure 1 could prove highly instructive. Something like a 'data life cycle best practices cheat sheet' ;) 

If you (the authors) have any questions regarding this review, please do not hesitate to contact me.

I have read this submission. I believe that I have an appropriate level of expertise to confirm that it is of an acceptable scientific standard, however I have significant reservations, as outlined above.
